# Fragranced consumer products: effects on asthmatics

**DOI:** 10.1007/s11869-017-0536-2

**Published:** 2017-12-11

**Authors:** Anne Steinemann

**Affiliations:** 1 Department of Infrastructure Engineering, Melbourne School of Engineering, The University of Melbourne, Melbourne, VIC 3010 Australia; 2 College of Science and Engineering, James Cook University, Townsville, QLD 4811 Australia; 3 Climate, Atmospheric Sciences, and Physical Oceanography, Scripps Institution of Oceanography, University of California, San Diego, La Jolla, CA 92093 USA

**Keywords:** Asthma, Fragranced consumer products, Indoor air quality, Fragrance, Health effects, Volatile organic compounds, Semi-volatile organic compounds

## Abstract

Fragranced consumer products, such as cleaning supplies, air fresheners, and personal care products, can emit a range of air pollutants and trigger adverse health effects. This study investigates the prevalence and types of effects of fragranced products on asthmatics in the American population. Using a nationally representative sample (*n* = 1137), data were collected with an on-line survey of adults in the USA, of which 26.8% responded as being medically diagnosed with asthma or an asthma-like condition. Results indicate that 64.3% of asthmatics report one or more types of adverse health effects from fragranced products, including respiratory problems (43.3%), migraine headaches (28.2%), and asthma attacks (27.9%). Overall, asthmatics were more likely to experience adverse health effects from fragranced products than non-asthmatics (prevalence odds ratio [POR] 5.76; 95% confidence interval [CI] 4.34–7.64). In particular, 41.0% of asthmatics report health problems from air fresheners or deodorizers, 28.9% from scented laundry products coming from a dryer vent, 42.3% from being in a room cleaned with scented products, and 46.2% from being near someone wearing a fragranced product. Of these effects, 62.8% would be considered disabling under the definition of the Americans with Disabilities Act. Yet 99.3% of asthmatics are exposed to fragranced products at least once a week. Also, 36.7% cannot use a public restroom if it has an air freshener or deodorizer, and 39.7% would enter a business but then leave as quickly as possible due to air fresheners or some fragranced product. Further, 35.4% of asthmatics have lost workdays or a job, in the past year, due to fragranced product exposure in the workplace. More than twice as many asthmatics would prefer that workplaces, health care facilities and health care professionals, hotels, and airplanes were fragrance-free rather than fragranced. Results from this study point to relatively simple and cost-effective ways to reduce exposure to air pollutants and health risks for asthmatics by reducing their exposure to fragranced products.

**Electronic supplementary material:**

The online version of this article (10.1007/s11869-017-0536-2) contains supplementary material, which is available to authorized users.

## Introduction

Fragranced consumer products pervade society and emit numerous volatile organic compounds, such as limonene, alpha-pinene, beta-pinene, acetaldehyde, and formaldehyde (Steinemann 2015; Nazaroff and Weschler 2004), and semi-volatile organic compounds, such as musks and phthalates (Weschler 2009; Just et al. 2010). However, ingredients in fragranced products are exempt from full disclosure on product labels or safety data sheets (Steinemann 2015), limiting awareness of potential emissions and exposures. Fragranced products have been associated with a range of adverse health effects including work-related asthma (Weinberg et al. 2017), asthmatic exacerbations (Kumar et al. 1995; Millqvist and Löwhagen 1996), respiratory difficulties (Caress and Steinemann 2009), mucosal symptoms (Elberling et al. 2005), migraine headaches (Kelman 2004), and contact dermatitis (Rastogi et al. 2007; Johansen 2003), as well as neurological, cardiovascular, cognitive, musculoskeletal, and immune system problems (Steinemann 2016).

This article investigates specifically the effects of exposure to fragranced products on asthmatics in the US population. In addition to health impacts, it also investigates societal access, preferences for fragrance-free environments, awareness of fragranced product emissions, and implications for air quality and health. It compares results from the sub-population of asthmatics with non-asthmatics, as well as with the general US population, as reported in Steinemann (2016). The study provides important data on the extent and severity of the problem, pointing to opportunities to reduce the adverse health, economic, and societal effects by reducing exposure to fragranced products.

## Methods

A nationally representative on-line survey was conducted of the US population, representative of age, gender, and region (*n* = 1137, confidence limit = 95%, confidence interval = 3%). The survey drew upon a large web-based US panel (over 5,000,000 people) held by Survey Sampling International, using randomized participant recruitment (SSI 2016). The survey instrument was developed and tested over a two-year period before full implementation in June 2016. The survey response rate was 95% (responses to panel recruitment 1201; screen-outs 13; drop-outs 46; completes 1137), and all responses were anonymous. The research study received ethics approval from the University of Melbourne. Details on the survey methodology are provided as a supplemental document.

This article extends and deepens the general population study of Steinemann (2016) by analyzing specifically the effects on asthmatics and compared to non-asthmatics and the general population. Of the general population surveyed, 26.8% responded as being medically diagnosed with either asthma (15.2%, *n* = 173) or an asthma-like condition (12.5%, *n* = 142) or both (26.8%, *n* = 305). For the purposes of the article, the sub-population of “asthmatics” will be those medically diagnosed with asthma, an asthma-like condition, or both; the sub-population of “non-asthmatics” will be those in the general population other than asthmatics.

Survey questions investigated use and exposure to fragranced products, both from one’s own use and from others’ use, exposure contexts and products, health effects related to exposures, impacts of fragrance exposure in the workplace and in society, awareness of fragranced product ingredients and labeling, preferences for fragrance-free environments and policies, and demographic information.

Specific exposure contexts included air fresheners or deodorizers used in public restrooms and other environments, scented laundry products coming from a dryer vent, being in a room after it was cleaned with scented cleaning products, being near someone wearing a fragranced product, entering a business with the scent of fragranced products, fragranced soap used in public restrooms, and ability to access environments that used fragranced products.

Fragranced products were categorized as follows: (a) air fresheners and deodorizers (e.g., sprays, solids, oils, disks); (b) personal care products (e.g., soaps, hand sanitizer, lotions, deodorant, sunscreen, shampoos); (c) cleaning supplies (e.g., all-purpose cleaners, disinfectants, dishwashing soap); (d) laundry products (e.g., detergents, fabric softeners, dryer sheets); (e) household products (e.g., scented candles, restroom paper, trash bags, baby products); (f) fragrance (e.g., perfume, cologne, after-shave); and (g) other.

Health effects were categorized as follows: (a) migraine headaches; (b) asthma attacks; (c) neurological problems (e.g., dizziness, seizures, head pain, fainting, loss of coordination); (d) respiratory problems (e.g., difficulty breathing, coughing, shortness of breath); (e) skin problems (e.g., rashes, hives, red skin, tingling skin, dermatitis); (f) cognitive problems (e.g., difficulties thinking, concentrating, or remembering); (g) mucosal symptoms (e.g., watery or red eyes, nasal congestion, sneezing); (h) immune system problems (e.g., swollen lymph glands, fever, fatigue); (i) gastrointestinal problems (e.g., nausea, bloating, cramping, diarrhea); (j) cardiovascular problems (e.g., fast or irregular heartbeat, jitteriness, chest discomfort); (k) musculoskeletal problems (e.g., muscle or joint pain, cramps, weakness); and (j) other. Categories were derived from prior studies of fragranced products and health effects (Caress and Steinemann 2009; Miller and Prihoda 1999) and pre-tested before full survey implementation.

## Results

Main findings are presented in this section, and full results for asthmatics, non-asthmatics, and the general population are provided as supplemental documentation. Demographic information is provided in Table 1.

**Table 1:** Demographic information

	Asthmatics		Non-asthmatics		General population
	*N*	*N*	*N* % of column total	*N* % of general population row	*N* % of column total
	% of column total	% of general population row			
Total	305	305	832	832	1137
	100.0%	26.8%	100.0%	73.2%	100.0%
Male/female					
All males	136	136	389	389	525
	44.6%	25.9%	46.8%	74.1%	46.2%
All females	169	169	443	443	612
	55.4%	27.6%	53.2%	72.4%	53.8%
Gender–age					
Male 18–24	16	16	31	31	47
	5.2%	34.0%	3.7%	66.0%	4.1%
Male 25–34	36	36	94	94	130
	11.8%	27.7%	11.3%	72.3%	11.4%
Male 35–44	42	42	94	94	136
	13.8%	30.9%	11.3%	69.1%	12.0%
Male 45–54	30	30	78	78	108
	9.8%	27.8%	9.4%	72.2%	9.5%
Male 55–65	12	12	92	92	104
	3.9%	11.5%	11.1%	88.5%	9.1%
Female 18–24	26	26	52	52	78
	8.5%	33.3%	6.3%	66.7%	6.9%
Female 25–34	40	40	95	95	135
	13.1%	29.6%	11.4%	70.4%	11.9%
Female 35–44	43	43	112	112	155
	14.1%	27.7%	13.5%	72.3%	13.6%
Female 45–54	41	41	103	103	144
	13.4%	28.5%	12.4%	71.5%	12.7%
Female 55–65	19	19	81	81	100
	6.2%	19.0%	9.7%	81.0%	8.8%

### Fragranced product exposure

Among asthmatics, 99.0% are exposed to fragranced products at least once a week, from their own use (71.1% air fresheners and deodorizers; 85.9% personal care products; 78.4% cleaning supplies; 81.3% laundry products; 76.7% household products; 67.5% fragrance; 3.6% other). Further, 94.8% are exposed to fragranced products at least once a week, from others’ use. Combined, 99.3% of asthmatics are exposed to fragranced products through their own use, others’ use, or both. Among non-asthmatics, 98.1% are exposed to fragranced products at least once a week from their own use, 91.1% from others’ use, and 98.9% from either or both. Thus, asthmatics are more likely to be exposed to fragranced products, from their own use and others’ use and both, than non-asthmatics (POR, 1.66; 95% CI, 0.36–7.71).

### Adverse health effects

Among asthmatics, 64.3% reported one or more types of adverse health effects from exposure to one or more types of fragranced products (43.3% respiratory problems; 27.2% mucosal symptoms; 28.2% migraine headaches; 19.0% skin problems; 27.9% asthma attacks; 15.1% neurological problems; 14.1% cognitive problems; 12.1% gastrointestinal problems; 9.8% cardiovascular problems; 11.1% immune system problems; 9.5% musculoskeletal problems; and 1.3% other). Among non-asthmatics, 23.8% reported one or more types of adverse health effects from exposure to one or more types of fragranced products (see Table 2). Thus, among all types of health effects (excepting asthma attacks), asthmatics are more likely to be affected than non-asthmatics (POR 5.76; 95% CI, 4.34–7.64).

**Table 2:** Frequency and types of adverse health effects reported from exposure to fragranced consumer products

	Asthmatics	Non-asthmatics	General population
	305	832	1137
	26.8%	73.2%	100.0%
Migraine headaches	86	93	179
	28.2%	11.2%	15.7%
Asthma attacks	85	6	91
	27.9%	0.7%	8.0%
Neurological problems	46	36	82
	15.1%	4.3%	7.2%
Respiratory problems	132	79	211
	43.3%	9.5%	18.6%
Skin problems	58	63	121
	19.0%	7.6%	10.6%
Cognitive problems	43	23	66
	14.1%	2.8%	5.8%
Mucosal symptoms	83	101	184
	27.2%	12.1%	16.2%
Immune system problems	34	11	45
	11.1%	1.3%	4.0%
Gastrointestinal problems	37	26	63
	12.1%	3.1%	5.5%
Cardiovascular problems	30	20	50
	9.8%	2.4%	4.4%
Musculoskeletal problems	29	14	43
	9.5%	1.7%	3.8%
Other	4	15	19
	1.3%	1.8%	1.7%
Total	196	198	394
(One or more health problems)	*64.3%*	*23.8%*	*34.7%*

Of the 64.3% of asthmatics reporting adverse health effects from fragranced products, proportionately more males report adverse effects than females, relative to non-asthmatics (asthmatic 52.0% female, 48.0% male; non-asthmatic 60.1% female, 39.9% male) (POR 1.39; 95% CI, 0.93–2.97) (see Table 3). Among all age groups, proportionately more asthmatics in age group 25–34 report adverse effects relative to non-asthmatics (asthmatic 69.7%; non-asthmatic 23.3%) (POR 7.59; 95% CI, 4.19–13.76). Among all gender and age groups, proportionately more males age 25–34 report adverse effects relative to non-asthmatics (asthmatic 83.3%; non-asthmatic 18.1%) (POR 22.65; 95% CI, 8.15–62.92).

**Table 3:** Demographic information for individuals reporting adverse effects from exposure to fragranced products

	Asthmatics		Non-asthmatics		General population	
	*N* % of column total	*N* % of asthmatics row, Table 1	*N* % of column total	*N* % of non-asthmatics row, Table 1	*N* % of column total	*N* % of general population row, Table 1
Total	196	196	198	198	394	394
	100.0%	64.3%	100.0%	23.8%	100.0%	34.7%
Male/female						
All males	94	94	79	79	173	173
	48.0%	69.1%	39.9%	20.3%	43.9%	33.0%
All females	102	102	119	119	221	221
	52.0%	60.4%	60.1%	26.9%	56.1%	36.1%
Gender–age						
Male 18–24	8	8	6	6	14	14
	4.1%	50.0%	3.0%	19.4%	3.6%	29.8%
Male 25–34	30	30	17	17	47	47
	15.3%	83.3%	8.6%	18.1%	11.9%	36.2%
Male 35–44	31	31	24	24	55	55
	15.8%	73.8%	12.1%	25.5%	14.0%	40.4%
Male 45–54	17	17	15	15	32	32
	8.7%	56.7%	7.6%	19.2%	8.1%	29.6%
Male 55–65	8	8	17	17	25	25
	4.1%	66.7%	8.6%	18.5%	6.3%	24.0%
Female 18–24	12	12	8	8	20	20
	6.1%	46.2%	4.0%	15.4%	5.1%	25.6%
Female 25–34	23	23	27	27	50	50
	11.7%	57.5%	13.6%	28.4%	12.7%	37.0%
Female 35–44	28	28	33	33	61	61
	14.3%	65.1%	16.7%	29.5%	15.5%	39.4%
Female 45–54	27	27	26	26	53	53
	13.8%	65.9%	13.1%	25.2%	13.5%	36.8%
Female 55–65	12	12	25	25	37	37
	6.1%	63.2%	12.6%	30.9%	9.4%	37.0%

### Specific exposure contexts

Air fresheners and deodorizers were associated with health problems for 41.0% of asthmatics (54.4% respiratory problems, 39.2% asthma attacks, 29.6% mucosal symptoms, 36.8% migraine headaches, 15.2% neurological problems, 26.4% skin problems, and others), and for 12.9% of non-asthmatics (see Table 4). Thus, asthmatics were more likely to experience adverse effects from air fresheners than non-asthmatics (POR 4.71; 95% CI, 3.47–6.39).

**Table 4:** Frequency and types of health problems experienced by asthmatics, non-asthmatics, and the general population from exposure to four types of fragranced consumer products

	Air fresheners or deodorizers			Scented laundry products			Scented cleaning products			Fragranced person		
	Asth	Non-asth	Gen Pop	Asth	Non-asth	Gen Pop	Asth	Non-asth	Gen Pop	Asth	Non-asth	Gen Pop
Health problem	125	107	232	88	54	142	129	95	224	141	127	268
	*41.0%*	*12.9%*	*20.4%*	*28.9%*	*6.5%*	*12.5%*	*42.3%*	*11.4%*	*19.7%*	*46.2%*	*15.3%*	*23.6%*
Migraines	46	36	82	24	13	37	42	33	75	45	51	96
	36.8%	33.6%	35.3%	27.3%	24.1%	26.1%	32.6%	34.7%	33.5%	31.9%	40.2%	35.8%
Asthma attacks	49	4	53	27	1	28	42	4	46	41	3	44
	39.2%	3.7%	22.8%	30.7%	1.9%	19.7%	32.6%	4.2%	20.5%	29.1%	2.4%	16.4%
Neurological	19	17	36	16	8	24	28	19	47	27	14	41
	15.2%	15.9%	15.5%	18.2%	14.8%	16.9%	21.7%	20.0%	21.0%	19.1%	11.0%	15.3%
Respiratory	68	40	108	34	12	46	67	42	109	77	41	118
	54.4%	37.4%	46.6%	38.6%	22.2%	32.4%	51.9%	44.2%	48.7%	54.6%	32.3%	44.0%
Skin	33	32	65	22	19	41	25	20	45	24	15	39
	26.4%	29.9%	28.0%	25.0%	35.2%	28.9%	19.4%	21.1%	20.1%	17.0%	11.8%	14.6%
Cognitive	15	16	31	9	6	15	21	10	31	21	9	30
	12.0%	15.0%	13.4%	10.2%	11.1%	10.6%	16.3%	10.5%	13.8%	14.9%	7.1%	11.2%
Mucosal	37	49	86	27	21	48	35	48	83	40	58	98
	29.6%	45.8%	37.1%	30.7%	38.9%	33.8%	27.1%	50.5%	37.1%	28.4%	45.7%	36.6%
Immune system	16	5	21	16	3	19	18	5	23	17	2	19
	12.8%	4.7%	9.1%	18.2%	5.6%	13.4%	14.0%	5.3%	10.3%	12.1%	1.6%	7.1%
Gastrointestinal	18	13	31	20	9	29	17	15	32	21	10	31
	14.4%	12.1%	13.4%	22.7%	16.7%	20.4%	13.2%	15.8%	14.3%	14.9%	7.9%	11.6%
Cardiovascular	18	12	30	11	4	15	16	10	26	15	5	20
	14.4%	11.2%	12.9%	12.5%	7.4%	10.6%	12.4%	10.5%	11.6%	10.6%	3.9%	7.5%
Musculoskeletal	19	8	27	21	2	23	13	10	23	15	2	17
	15.2%	7.5%	11.6%	23.9%	3.7%	16.2%	10.1%	10.5%	10.3%	10.6%	1.6%	6.3%
Other	2	6	8	1	3	4	2	2	4	2	5	7

Scented laundry products coming from a dryer vent were associated with health problems for 28.9% of asthmatics (38.6% respiratory problems, 30.7% asthma attacks, 30.7% mucosal symptoms, 27.3% migraine headaches, 18.2% neurological problems, 25.0% skin problems, and others), and for 6.5% of non-asthmatics (see Table 4). Thus, asthmatics were more likely to experience adverse effects from scented laundry products coming from a dryer vent than non-asthmatics (POR 5.84; 95% CI, 4.03–8.46).

Being in a room after it has been cleaned with scented products was associated with health problems for 42.3% of asthmatics (51.9% respiratory problems, 32.6% asthma attacks, 27.1% mucosal symptoms, 32.6% migraine headaches, 21.7% neurological problems, 19.4% skin problems, and others), and for 11.4% of non-asthmatics (see Table 4). Thus, asthmatics were more likely to experience adverse effects from being in a room after it has been cleaned with scented products than non-asthmatics (POR 5.69; 95% CI, 4.16–7.77).

Being near someone wearing a fragranced product was associated with health problems for 46.2% of asthmatics (54.6% respiratory problems, 29.1% asthma attacks, 28.4% mucosal symptoms, 31.9% migraine headaches, 19.1% neurological problems, 17.0% skin problems, and others), and 15.3% of non-asthmatics (see Table 4). Thus, asthmatics were more likely to experience adverse effects from being near someone wearing a fragranced product than non-asthmatics (POR 4.77; 95% CI, 3.56–6.40).

Exposure to fragranced products can trigger disabling health effects, according to criteria from the Americans with Disabilities Act (ADA 1990): "Do any of these health problems substantially limit one or more major life activities, such as seeing, hearing, eating, sleeping, walking, standing, lifting, bending, speaking, breathing, learning, reading, concentrating, thinking, communicating, or working, for you personally?" Among asthmatics reporting health problems, 62.8% reported that the severity of the health effect from fragranced product exposure was potentially disabling. Thus, asthmatics were more likely to report disabling health effects from fragranced products than non-asthmatics (POR 7.13; 95% CI, 5.11–9.95).

### Ingredient disclosure and product claims

Among asthmatics, 41.3% were not aware that a “fragrance” in a product is typically a chemical mixture of several dozen to several hundred chemicals, 57.4% were not aware that fragrance chemicals do not need to be fully disclosed on the product label or material safety data sheet, and 58.0% were not aware that fragranced products typically emit hazardous air pollutants such as formaldehyde. Further, 64.3% of asthmatics, and 75.7% of non-asthmatics, were not aware that even so-called natural, green, and organic fragranced products typically emit hazardous air pollutants (28.9% of asthmatics and 15.7% of non-asthmatics were aware). However, 60.3% of asthmatics, and 60.1% of non-asthmatics, would not still use a fragranced product if they knew it emitted hazardous air pollutants.

### Societal and workplace effects

Fragranced products can also present barriers for asthmatics in public places and the workplace. Among asthmatics, 36.7% are prevented from using the restrooms in a public place, because of the presence of an air freshener, deodorizer, or scented product. Also, 28.9% are prevented from washing their hands with soap in a public place, if the soap is fragranced. Further, 43.9% are prevented from going to some place because they would be exposed to a fragranced product that would make them sick. Notably, 39.7% report that if they enter a business, and smell air fresheners or some fragranced product, they want to leave as quickly as possible.

Significantly, 35.4% of asthmatics, and 7.7% of non-asthmatics, have become sick, lost workdays, or lost a job, in the past 12 months, due to fragranced products in their work environment. Thus, asthmatics were more likely to have lost workdays or lost a job due to illness from fragranced products in their work environment than non-asthmatics (POR 6.58; 95% CI, 4.65–9.30).

Fragrance-free policies receive a strong majority of support. Among asthmatics, 66.2% would be supportive of a fragrance-free policy in the workplace (compared to 16.1% that would not). Thus, more than four times as many asthmatics would prefer a fragrance-free workplace than fragranced. Also, 72.1% of asthmatics would prefer that health care facilities and health care professionals be fragrance-free (compared to 14.8% that would not). Thus, nearly five times as many asthmatics would prefer fragrance-free health care facilities and professionals than fragranced.

Among non-asthmatics, 48.3% would support a fragrance-free workplace (compared with 21.0% that would not), and among the general population, 53.1% would support a fragrance-free workplace (compared with 19.7% that would not). Thus, regardless of population, fragrance-free workplaces receive more than twice as many in support as not.

Asthmatics also strongly prefer fragrance-free airplanes and hotels. If given a choice between flying on an airplane that pumped scented air throughout the passenger cabin, or did not pump scented air throughout the passenger cabin, 63.6% of asthmatics would choose an airplane without scented air (compared to 24.9% with scented air). Similarly, if given a choice between staying in a hotel with fragranced air, or without fragranced air, 63.0% would choose a hotel without fragranced air (compared to 28.5% with fragranced air).

Among non-asthmatics, 57.6 and 52.9% would prefer fragrance-free airplanes and hotels, respectively (compared with 23.1 and 27.5% that would not) and among the general population, 59.2 and 55.6% would prefer fragrance-free airplanes and hotels, respectively (compared with 23.6 and 27.8% that would not). Thus, overall, more than twice as many asthmatics, as well as the general population, would prefer that airplanes and hotels were fragrance-free rather than fragranced.

## Discussion

Asthma is a serious and increasing health condition, affecting an estimated 25 million Americans, and costing an estimated $56 billion annually in medical expenses, missed school and work days, and premature deaths (CDCP 2017a). Nearly 12 million Americans had an asthma attack in 2015, many of which could have been prevented (CDCP 2017b).

Results from this study show that asthmatics are profoundly, adversely, and disproportionately affected by exposure to fragranced consumer products. While non-asthmatics are also affected, asthmatics are more likely to experience adverse health effects from exposure (POR 5.76; 95% CI 4.34–7.64).

Of particular concern are involuntary exposures to fragranced products, such as in health care facilities and workplaces. Asthmatics are prevented from accessing public toilets, businesses, and workplaces due to adverse health effects from fragranced products. Further, 35.4% have lost workdays or a job, in the past year, due to fragranced product exposure in the workplace. More than twice as many asthmatics would prefer that workplaces, health care facilities, health care professionals, airplanes, and hotels were fragrance-free than fragranced.

Limitations of the study include the following: (a) data were based on self-reports, although a well-established method for survey research; (b) all possible products and health effects were not included, although the low percentages for responses in the “other” category indicates the survey captured the primary products and effects; (c) product emissions and exposures were not measured directly; (d) the cross-sectional design of the study, while useful for determining prevalence, provides data that represent just one point in time, limiting the analysis of risk factors, temporal relationships between exposures and effects, and trends in prevalence, and (e) only adults (ages 18–65) were included in the survey, which overlooks the effects of fragranced products on children (such as in day care facilities and schools) and on seniors (such as in retirement communities and assisted living facilities).

Results of this study provide strong evidence that fragranced consumer products can harm health for both asthmatics and non-asthmatics, with asthmatics more affected. Understanding why these products are associated with a range of health problems is a critical topic that requires further research. Fragranced products emit a range of volatile and semi-volatile organic compounds, some of which are associated with adverse health effects, but virtually none of which need to be disclosed (Steinemann 2009, 2015), thus limiting scientific inquiry and public awareness of potential exposures to problematic compounds. A broader mechanistic framework is needed to understand which ingredients, or combinations of ingredients, could be associated with the adverse health outcomes reported in this study. In the meantime, a prudent and practical approach, and one that would provide direct and immediate benefits, would be to limit exposure to fragranced consumer products.

## Electronic supplementary material

ESM 1 (PDF 159 kb)

ESM 2 (PDF 69 kb)
