# Supplementary material for: Fragranced consumer products: effects on asthmatics
Source: Air Qual Atmos Health. 2017 Dec 11;11(1):3–9. doi: 10.1007/s11869-017-0536-2 (PMC5773620; doi:10.1007/s11869-017-0536-2)
Supplement: Supplementary file 1 — (PDF 159 kb) [file 11869_2017_536_MOESM1_ESM.pdf]

Table A

What is your gender?

	Asthmatic	Non-asthmatic	General population
Total	305	832	1137
	100.00%	100.00%	100.00%
Male	136	389	525
	44.60%	46.80%	46.20%
Female	169	443	612
	55.40%	53.20%	53.80%
Other	-	-	-
	-	-	-

Table B

What is your age?

	Asthmatic	Non-asthmatic	General population
Total	305	832	1137
	100.00%	100.00%	100.00%
18-24 (21)	42	83	125
	13.80%	10.00%	11.00%
25-34 (29.5)	76	189	265
	24.90%	22.70%	23.30%
35-44 (39.5)	85	206	291
	27.90%	24.80%	25.60%
45-54 (49.5)	71	181	252
	23.30%	21.80%	22.20%
55-65 (60)	31	173	204
	10.20%	20.80%	17.90%
65 (65)	-	-	-
	-	-	-

Table C

USA Region

	Asthmatic	Non-asthmatic	General population
Total	305	832	1137
	100.00%	100.00%	100.00%
Northeast	58	149	207
	19.00%	17.90%	18.20%
Midwest	62	184	246
	20.30%	22.10%	21.60%
South	105	317	422
	34.40%	38.10%	37.10%
West	80	182	262
	26.20%	21.90%	23.00%
SUM	305	832	1137
	100.00%	100.00%	100.00%

Table D

Which fragranced products are you exposed to, at least once a week, from your own use?

	Asthmatic	Non-asthmatic	General population
Total	305	832	1137
	100.00%	100.00%	100.00%
Yes (net)	302	816	1118
	99.00%	98.10%	98.30%
Air fresheners and deodorizers (e.g., sprays, solids, oils, disks)	217	611	828
	71.10%	73.40%	72.80%
Personal care products (e.g., soaps, hand sanitizer, lotions, deodorant, sunscreen, shampoos)	262	748	1010
	85.90%	89.90%	88.80%
Cleaning supplies (e.g., all-purpose cleaners, disinfectants, dishwashing soap)	239	670	909
	78.40%	80.50%	79.90%
Laundry products (e.g., detergents, fabric softeners, dryer sheets)	248	708	956
	81.30%	85.10%	84.10%
Household products (e.g., scented candles, toilet paper, trash bags, baby products)	234	642	876
	76.70%	77.20%	77.00%
Fragrance (e.g., perfume, cologne, after-shave)	206	592	798
	67.50%	71.20%	70.20%
Other	11	23	34
	3.60%	2.80%	3.00%
None	3	16	19
	1.00%	1.90%	1.70%

Table E

Which fragranced products are you exposed to, at least once a week, from others' use?

	Asthmatic	Non-asthmatic	General population
Total	305	832	1137
	100.00%	100.00%	100.00%
Yes (net)	289	758	1047
	94.80%	91.10%	92.10%
Air fresheners and deodorizers (e.g., sprays, solids, oils, disks)	176	482	658
	57.70%	57.90%	57.90%
Personal care products (e.g., soaps, hand sanitizer, lotions, deodorant, sunscreen, shampoos)	212	539	751
	69.50%	64.80%	66.10%
Cleaning supplies (e.g., all-purpose cleaners, disinfectants, dishwashing soap)	163	459	622
	53.40%	55.20%	54.70%
Laundry products (e.g., detergents, fabric softeners, dryer sheets)	145	394	539
	47.50%	47.40%	47.40%
Household products (e.g., scented candles, toilet paper, trash bags, baby products)	156	438	594
	51.10%	52.60%	52.20%
Fragrance (e.g., perfume, cologne, after-shave)	217	564	781
	71.10%	67.80%	68.70%
Other	14	22	36
	4.60%	2.60%	3.20%
None	16	74	90
	5.20%	8.90%	7.90%

Table F

Respondents exposed to products from Own Use, Others' Use, or Both

	Asthmatic	Non-asthmatic	General population
Total	305<br>100.00%	832<br>100.00%	1137<br>100.00%
Yes (net)	303<br>99.30%	823<br>98.90%	1126<br>99.00%
Air fresheners and deodorizers<br>(e.g., sprays, solids, oils, disks)	238<br>78.00%	661<br>79.40%	899<br>79.10%
Personal care products<br>(e.g., soaps, hand sanitizer, lotions<br>deodorant, sunscreen, shampoos)	276<br>90.50%	772<br>92.80%	1048<br>92.20%
Cleaning supplies<br>(e.g., all-purpose cleaners,<br>disinfectants, dishwashing soap)	259<br>84.90%	721<br>86.70%	980<br>86.20%
Laundry products<br>(e.g., detergents, fabric softeners,<br>dryer sheets)	263<br>86.20%	742<br>89.20%	1005<br>88.40%
Household products<br>(e.g., scented candles, toilet paper,<br>trash bags, baby products)	248<br>81.30%	688<br>82.70%	936<br>82.30%
Fragrance<br>(e.g., perfume, cologne,<br>after-shave)	245<br>80.30%	681<br>81.90%	926<br>81.40%
Other	18<br>5.90%	32<br>3.80%	50<br>4.40%
None	17<br>5.60%	81<br>9.70%	98<br>8.60%

Table G

Q2: Do you experience any health problems when exposed to air fresheners or deodorizers?

	Asthmatic	Non-asthmatic	General population
Total	305	832	1137
	100.00%	100.00%	100.00%
Yes	125	107	232
	41.00%	12.90%	20.40%
No	148	643	791
	48.50%	77.30%	69.60%
Don't know/not sure	29	80	109
	9.50%	9.60%	9.60%
Decline to answer	3	2	5
	1.00%	0.20%	0.40%
SUM	305	832	1137
	100.00%	100.00%	100.00%

Table H

Which of the following health problems do you (...)?

Base: Respondents who experienced below health problems when exposed to air fresheners or deodorizers

	Asthmatic	Non-asthmatic	General population
Total	125	107	232
	100.00%	100.00%	100.00%
Migraine headaches	46	36	82
	36.80%	33.60%	35.30%
Asthma attacks	49	4	53
	39.20%	3.70%	22.80%
Neurological problems (e.g., dizziness, seizures,	19	17	36
head pain, fainting, loss of coordination)	15.20%	15.90%	15.50%
Respiratory problems (e.g., difficulty breathing,	68	40	108
coughing, shortness of breath)	54.40%	37.40%	46.60%
Skin problems (e.g., rashes, hives, red skin,	33	32	65
tingling skin, dermatitis)	26.40%	29.90%	28.00%
Cognitive problems (e.g., difficulties thinking,	15	16	31
concentrating, or remembering)	12.00%	15.00%	13.40%
Mucosal symptoms (e.g., watery or red eyes, nasal	37	49	86
congestion, sneezing)	29.60%	45.80%	37.10%
Immune system problems (e.g., swollen lymph	16	5	21
glands, fever, fatigue)	12.80%	4.70%	9.10%
Gastrointestinal problems (e.g., nausea, bloating,	18	13	31
cramping, diarrhea)	14.40%	12.10%	13.40%
Cardiovascular problems (e.g., fast or irregular	18	12	30
heartbeat, jitteriness, chest discomfort)	14.40%	11.20%	12.90%
Musculoskeletal problems (e.g., muscle or joint	19	8	27
pain, cramps, weakness)	15.20%	7.50%	11.60%
Other	2	6	8
	1.60%	5.60%	3.40%
SUM	340	238	578
	272.00%	222.40%	249.10%

Table I

Q3: Do you experience any health problems from the scent of laundry products coming from a dryer vent?

	Asthmatic	Non-asthmatic	General population
Total	305	832	1137
	100.00%	100.00%	100.00%
Yes	88	54	142
	28.90%	6.50%	12.50%
No	193	713	906
	63.30%	85.70%	79.70%
Don't know/not sure	24	64	88
	7.90%	7.70%	7.70%
Decline to answer	-	1	1
	-	0.10%	0.10%
SUM	305	832	1137
	100.00%	100.00%	100.00%

Table J

Which of the following health problems do you (...)?

Base: Respondents who experienced below health problems from the scent of laundry products coming from a dryer vent

	Asthmatic	Non-asthmatic	General population
Total	88	54	142
	100.00%	100.00%	100.00%
Migraine headaches	24	13	37
	27.30%	24.10%	26.10%
Asthma attacks	27	1	28
	30.70%	1.90%	19.70%
Neurological problems (e.g., dizziness, seizures,	16	8	24
head pain, fainting, loss of coordination)	18.20%	14.80%	16.90%
Respiratory problems (e.g., difficulty breathing,	34	12	46
coughing, shortness of breath)	38.60%	22.20%	32.40%
Skin problems (e.g., rashes, hives, red skin,	22	19	41
tingling skin, dermatitis)	25.00%	35.20%	28.90%
Cognitive problems (e.g., difficulties thinking,	9	6	15
concentrating, or remembering)	10.20%	11.10%	10.60%
Mucosal symptoms (e.g., watery or red eyes, nasal	27	21	48
congestion, sneezing)	30.70%	38.90%	33.80%
Immune system problems (e.g., swollen lymph	16	3	19
glands, fever, fatigue)	18.20%	5.60%	13.40%
Gastrointestinal problems (e.g., nausea, bloating,	20	9	29
cramping, diarrhea)	22.70%	16.70%	20.40%
Cardiovascular problems (e.g., fast or irregular	11	4	15
heartbeat, jitteriness, chest discomfort)	12.50%	7.40%	10.60%
Musculoskeletal problems (e.g., muscle or joint	21	2	23
pain, cramps, weakness)	23.90%	3.70%	16.20%
Other	1	3	4
	1.10%	5.60%	2.80%
SUM	228	101	329
	259.10%	187.00%	231.70%

Table K

Q4: Do you experience any health problems from being in a room after it has been cleaned with scented products?

	Asthmatic	Non-asthmatic	General population
Total	305	832	1137
	100.00%	100.00%	100.00%
Yes	129	95	224
	42.30%	11.40%	19.70%
No	157	682	839
	51.50%	82.00%	73.80%
Don't know/not sure	19	54	73
	6.20%	6.50%	6.40%
Decline to answer	-	1	1
	-	0.10%	0.10%
SUM	305	832	1137
	100.00%	100.00%	100.00%

Table L

Which of the following health problems do you (...)?

Base: Respondents who experienced below health problems from being in a room after it has been cleaned with scented products

	Asthmatic	Non-asthmatic	General population
Total	129	95	224
	100.00%	100.00%	100.00%
Migraine headaches	42	33	75
	32.60%	34.70%	33.50%
Asthma attacks	42	4	46
	32.60%	4.20%	20.50%
Neurological problems (e.g., dizziness, seizures,	28	19	47
head pain, fainting, loss of coordination)	21.70%	20.00%	21.00%
Respiratory problems (e.g., difficulty breathing,	67	42	109
coughing, shortness of breath)	51.90%	44.20%	48.70%
Skin problems (e.g., rashes, hives, red skin,	25	20	45
tingling skin, dermatitis)	19.40%	21.10%	20.10%
Cognitive problems (e.g., difficulties thinking,	21	10	31
concentrating, or remembering)	16.30%	10.50%	13.80%
Mucosal symptoms (e.g., watery or red eyes, nasal	35	48	83
congestion, sneezing)	27.10%	50.50%	37.10%
Immune system problems (e.g., swollen lymph	18	5	23
glands, fever, fatigue)	14.00%	5.30%	10.30%
Gastrointestinal problems (e.g., nausea, bloating,	17	15	32
cramping, diarrhea)	13.20%	15.80%	14.30%
Cardiovascular problems (e.g., fast or irregular	16	10	26
heartbeat, jitteriness, chest discomfort)	12.40%	10.50%	11.60%
Musculoskeletal problems (e.g., muscle or joint	13	10	23
pain, cramps, weakness)	10.10%	10.50%	10.30%
Other	2	2	4
	1.60%	2.10%	1.80%
SUM	326	218	544
	252.70%	229.50%	242.90%

Table M

Q5: Do you experience any health problems from being near someone who is wearing a fragranced product?

	Asthmatic	Non-asthmatic	General population
Total	305	832	1137
	100.00%	100.00%	100.00%
Yes	141	127	268
	46.20%	15.30%	23.60%
No	145	654	799
	47.50%	78.60%	70.30%
Don't know/not sure	19	49	68
	6.20%	5.90%	6.00%
Decline to answer	-	2	2
	-	0.20%	0.20%
SUM	305	832	1137
	100.00%	100.00%	100.00%

Table N

Which of the following health problems do you (...)?

Base: Respondents who experienced below health problems from being near someone who is wearing a fragranced product

	Asthmatic	Non-asthmatic	General population
Total	141	127	268
	100.00%	100.00%	100.00%
Migraine headaches	45	51	96
	31.90%	40.20%	35.80%
Asthma attacks	41	3	44
	29.10%	2.40%	16.40%
Neurological problems (e.g., dizziness, seizures,	27	14	41
head pain, fainting, loss of coordination)	19.10%	11.00%	15.30%
Respiratory problems (e.g., difficulty breathing,	77	41	118
coughing, shortness of breath)	54.60%	32.30%	44.00%
Skin problems (e.g., rashes, hives, red skin,	24	15	39
tingling skin, dermatitis)	17.00%	11.80%	14.60%
Cognitive problems (e.g., difficulties thinking,	21	9	30
concentrating, or remembering)	14.90%	7.10%	11.20%
Mucosal symptoms (e.g., watery or red eyes, nasal	40	58	98
congestion, sneezing)	28.40%	45.70%	36.60%
Immune system problems (e.g., swollen lymph	17	2	19
glands, fever, fatigue)	12.10%	1.60%	7.10%
Gastrointestinal problems (e.g., nausea, bloating,	21	10	31
cramping, diarrhea)	14.90%	7.90%	11.60%
Cardiovascular problems (e.g., fast or irregular	15	5	20
heartbeat, jitteriness, chest discomfort)	10.60%	3.90%	7.50%
Musculoskeletal problems (e.g., muscle or joint	15	2	17
pain, cramps, weakness)	10.60%	1.60%	6.30%
Other	2	5	7
	1.40%	3.90%	2.60%
SUM	345	215	560
	244.70%	169.30%	209.00%

Table O

Q6: Do you experience any health problems from exposure to any type of fragranced product?

	Asthmatic	Non-asthmatic	General population
Total	305	832	1137
	100.00%	100.00%	100.00%
Yes	145	108	253
	47.50%	13.00%	22.30%
No	133	663	796
	43.60%	79.70%	70.00%
Don't know/not sure	27	60	87
	8.90%	7.20%	7.70%
Decline to answer	-	1	1
	-	0.10%	0.10%
SUM	305	832	1137
	100.00%	100.00%	100.00%

Table P

Which of the following health problems do you (...)?

Base: Respondents who experienced below health problems from exposure to any type of fragranced product

	Asthmatic	Non-asthmatic	General population
Total	145	108	253
	100.00%	100.00%	100.00%
Migraine headaches	44	54	98
	30.30%	50.00%	38.70%
Asthma attacks	53	2	55
	36.60%	1.90%	21.70%
Neurological problems (e.g., dizziness, seizures,	26	16	42
head pain, fainting, loss of coordination)	17.90%	14.80%	16.60%
Respiratory problems (e.g., difficulty breathing,	79	40	119
coughing, shortness of breath)	54.50%	37.00%	47.00%
Skin problems (e.g., rashes, hives, red skin,	38	20	58
tingling skin, dermatitis)	26.20%	18.50%	22.90%
Cognitive problems (e.g., difficulties thinking,	21	10	31
concentrating, or remembering)	14.50%	9.30%	12.30%
Mucosal symptoms (e.g., watery or red eyes, nasal	49	53	102
congestion, sneezing)	33.80%	49.10%	40.30%
Immune system problems (e.g., swollen lymph	20	4	24
glands, fever, fatigue)	13.80%	3.70%	9.50%
Gastrointestinal problems (e.g., nausea, bloating,	21	12	33
cramping, diarrhea)	14.50%	11.10%	13.00%
Cardiovascular problems (e.g., fast or irregular	13	5	18
heartbeat, jitteriness, chest discomfort)	9.00%	4.60%	7.10%
Musculoskeletal problems (e.g., muscle or joint	15	5	20
pain, cramps, weakness)	10.30%	4.60%	7.90%
Other	2	1	3
	1.40%	0.90%	1.20%
SUM	381	222	603
	262.80%	205.60%	238.30%

Table Q

Do any of these health problems substantially limit one or more major life activities, such as seeing, hearing, eating, sleeping, walking, standing, lifting, bending, speaking, breathing, learning, reading, concentrating, thinking, communicating, or working, for you personally? (ADA)

	Asthmatic	Non-asthmatic	General population
Total	196	198	394
	100.00%	100.00%	100.00%
Yes	123	72	195
	62.80%	36.40%	49.50%
No	61	114	175
	31.10%	57.60%	44.40%
Don't know/not sure	11	11	22
	5.60%	5.60%	5.60%
Decline to answer	1	1	2
	0.50%	0.50%	0.50%
SUM	196	198	394
	100.00%	100.00%	100.00%

Table R

Has a doctor or health care professional ever told you that you have asthma or an asthma-like condition?

	Asthmatic	Non-asthmatic	General population
Total	305	832	1137
	100.00%	100.00%	100.00%
Yes - asthma	173	-	173
	56.70%	-	15.20%
Yes - asthma-like condition	142	-	142
	46.60%	-	12.50%
No	-	811	811
	-	97.50%	71.30%
Don't know/not sure	-	19	19
	-	2.30%	1.70%
Decline to answer	-	2	2
	-	0.20%	0.20%
SUM	315	832	1147
	103.30%	100.00%	100.90%

Table S

"Yes" to Asthma or Asthma-Like Condition

	Asthmatic	Non-asthmatic	General population
Total	305	832	1137
	100.00%	100.00%	100.00%
Yes	305	-	305
	100.00%	-	26.80%

Table T

Have you ever been unable or reluctant to use the toilets in a public place, because of the presence of an air freshener, deodorizer, or scent

	Asthmatic	Non-asthmatic	General population
Total	305	832	1137
	100.00%	100.00%	100.00%
Yes	112	87	199
	36.70%	10.50%	17.50%
No	178	719	897
	58.40%	86.40%	78.90%
Neutral/not sure	15	25	40
	4.90%	3.00%	3.50%
Decline to answer	-	1	1
	-	0.10%	0.10%
SUM	305	832	1137
	100.00%	100.00%	100.00%

Table U

If you enter a business, and you smell air fresheners or some fragranced product, do you want to leave as quickly as possible?

	Asthmatic	Non-asthmatic	General population
Total	305	832	1137
	100.00%	100.00%	100.00%
Yes	121	108	229
	39.70%	13.00%	20.10%
No	147	640	787
	48.20%	76.90%	69.20%
Neutral/not sure	37	83	120
	12.10%	10.00%	10.60%
Decline to answer	-	1	1
	-	0.10%	0.10%
SUM	305	832	1137
	100.00%	100.00%	100.00%

Table V

Have you ever been unable or reluctant to wash your hands with soap in a public place, because you know or suspect that the soap is fragr

	Asthmatic	Non-asthmatic	General population
Total	305	832	1137
	100.00%	100.00%	100.00%
Yes	88	72	160
	28.90%	8.70%	14.10%
No	200	724	924
	65.60%	87.00%	81.30%
Neutral/not sure	16	34	50
	5.20%	4.10%	4.40%
Decline to answer	1	2	3
	0.30%	0.20%	0.30%
SUM	305	832	1137
	100.00%	100.00%	100.00%

Table W

Are you aware that a “fragrance” in a product is typically a chemical mixture of several dozen to several hundred chemicals?

	Asthmatic	Non-asthmatic	General population
Total	305	832	1137
	100.00%	100.00%	100.00%
Yes	159	340	499
	52.10%	40.90%	43.90%
No	126	402	528
	41.30%	48.30%	46.40%
Don't know/not sure	19	88	107
	6.20%	10.60%	9.40%
Decline to answer	1	2	3
	0.30%	0.20%	0.30%
SUM	305	832	1137
	100.00%	100.00%	100.00%

Table X

Are you aware that fragrance chemicals do not need to be fully disclosed on the product label or material safety data sheet?

	Asthmatic	Non-asthmatic	General population
Total	305	832	1137
	100.00%	100.00%	100.00%
Yes	109	184	293
	35.70%	22.10%	25.80%
No	175	560	735
	57.40%	67.30%	64.60%
Don't know/not sure	21	86	107
	6.90%	10.30%	9.40%
Decline to answer	-	2	2
	-	0.20%	0.20%
SUM	305	832	1137
	100.00%	100.00%	100.00%

Table Y

Are you aware that fragranced products typically emit hazardous air pollutants such as formaldehyde?

	Asthmatic	Non-asthmatic	General population
Total	305	832	1137
	100.00%	100.00%	100.00%
Yes	110	172	282
	36.10%	20.70%	24.80%
No	177	588	765
	58.00%	70.70%	67.30%
Don't know/not sure	17	69	86
	5.60%	8.30%	7.60%
Decline to answer	1	3	4
	0.30%	0.40%	0.40%
SUM	305	832	1137
	100.00%	100.00%	100.00%

Table Z

Are you aware that even so-called natural, green, and organic fragranced products typically emit hazardous air pollutants?

	Asthmatic	Non-asthmatic	General population
Total	305	832	1137
	100.00%	100.00%	100.00%
Yes	88	131	219
	28.90%	15.70%	19.30%
No	196	630	826
	64.30%	75.70%	72.60%
Don't know/not sure	20	68	88
	6.60%	8.20%	7.70%
Decline to answer	1	3	4
	0.30%	0.40%	0.40%
SUM	305	832	1137
	100.00%	100.00%	100.00%

Table AA

If you knew that a fragranced product emitted hazardous air pollutants, would you still use it?

	Asthmatic	Non-asthmatic	General population
Total	305	832	1137
	100.00%	100.00%	100.00%
Yes	76	115	191
	24.90%	13.80%	16.80%
No	184	500	684
	60.30%	60.10%	60.20%
Don't know/not sure	44	215	259
	14.40%	25.80%	22.80%
Decline to answer	1	2	3
	0.30%	0.20%	0.30%
SUM	305	832	1137
	100.00%	100.00%	100.00%

Table BB

Have you ever been prevented from going to some place because you would be exposed to a fragrance product that would make you sick?

	Asthmatic	Non-asthmatic	General population
Total	305	832	1137
	100.00%	100.00%	100.00%
Yes	134	124	258
	43.90%	14.90%	22.70%
No	156	663	819
	51.10%	79.70%	72.00%
Don't know/not sure	15	43	58
	4.90%	5.20%	5.10%
Decline to answer	-	2	2
	-	0.20%	0.20%
SUM	305	832	1137
	100.00%	100.00%	100.00%

Table CC

Has any exposure to fragranced products in your work environment caused you to become sick, lose work days, or lose a job?

	Asthmatic	Non-asthmatic	General population
Total	305	832	1137
	100.00%	100.00%	100.00%
Yes	108	64	172
	35.40%	7.70%	15.10%
No	178	732	910
	58.40%	88.00%	80.00%
Don't know/not sure	19	35	54
	6.20%	4.20%	4.70%
Decline to answer	-	1	1
	-	0.10%	0.10%
SUM	305	832	1137
	100.00%	100.00%	100.00%

Table DD

Would you be supportive of a fragrance-free policy in the workplace?

	Asthmatic	Non-asthmatic	General population
Total	305	832	1137
	100.00%	100.00%	100.00%
Yes	202	402	604
	66.20%	48.30%	53.10%
No	49	175	224
	16.10%	21.00%	19.70%
Neutral/not sure	53	251	304
	17.40%	30.20%	26.70%
Decline to answer	1	4	5
	0.30%	0.50%	0.40%
SUM	305	832	1137
	100.00%	100.00%	100.00%

Table EE

Would you prefer that health care facilities and health care professionals be fragrance-free?

	Asthmatic	Non-asthmatic	General population
Total	305	832	1137
	100.00%	100.00%	100.00%
Yes	220	403	623
	72.10%	48.40%	54.80%
No	45	210	255
	14.80%	25.20%	22.40%
Neutral/not sure	39	215	254
	12.80%	25.80%	22.30%
Decline to answer	1	4	5
	0.30%	0.50%	0.40%
SUM	305	832	1137
	100.00%	100.00%	100.00%

Table FF

Flying On An Airplane That Pumped / Did Not Pump Scented Air Throughout The Passenger Cabin, Which Would You Choose?

	Asthmatic	Non-asthmatic	General population
Total	305	832	1137
	100.00%	100.00%	100.00%
Airplane with scented air	76	192	268
	24.90%	23.10%	23.60%
Airplane without scented air	194	479	673
	63.60%	57.60%	59.20%
Neutral/not sure	35	154	189
	11.50%	18.50%	16.60%
Decline to answer	-	7	7
	-	0.80%	0.60%
SUM	305	832	1137
	100.00%	100.00%	100.00%

Table GG

Staying In A Hotel With / Without Fragranced Air, Which Would You Choose?

	Asthmatic	Non-asthmatic	General population
Total	305	832	1137
	100.00%	100.00%	100.00%
Hotel with fragranced air	87	229	316
	28.50%	27.50%	27.80%
Hotel without fragranced air	192	440	632
	63.00%	52.90%	55.60%
Neutral/not sure	26	159	185
	8.50%	19.10%	16.30%
Decline to answer	-	4	4
	-	0.50%	0.40%
SUM	305	832	1137
	100.00%	100.00%	100.00%

Table HH

What is your household annual income?

	Asthmatic	Non-asthmatic	General population
Total	305	832	1137
	100.00%	100.00%	100.00%
Less than $10,000 (10)	21	52	73
	6.90%	6.30%	6.40%
$10,000 - $49,999 (29.99)	102	268	370
	33.40%	32.20%	32.50%
$50,000 - $99,999 (74.99)	95	277	372
	31.10%	33.30%	32.70%
$100,000-$149,999 (124.99)	55	121	176
	18.00%	14.50%	15.50%
$150,000-$200,000 (175)	14	51	65
	4.60%	6.10%	5.70%
Over $200,000 (200)	8	20	28
	2.60%	2.40%	2.50%
Decline to answer	10	43	53
	3.30%	5.20%	4.70%

Table II

People Who Answer “Yes” To One Or More Of These Questions: Q2/Q3/Q4/Q5/Q6 (health problems from fragranced products).

	Asthmatic	Non-asthmatic	General population
Total	305	832	1137
	100.00%	100.00%	100.00%
Yes	196	198	394
	64.30%	23.80%	34.70%

Table JJ

Each type of health problem for each of these questions Q2/Q3/Q4/Q5/Q6.

	Asthmatic	Non-asthmatic	General population
Total	305	832	1137
	100.00%	100.00%	100.00%
Migraine headaches	86	93	179
	28.20%	11.20%	15.70%
Asthma attacks	85	6	91
	27.90%	0.70%	8.00%
Neurological problems (e.g., dizziness, seizures,	46	36	82
head pain, fainting, loss of coordination)	15.10%	4.30%	7.20%
Respiratory problems (e.g., difficulty breathing,	132	79	211
coughing, shortness of breath)	43.30%	9.50%	18.60%
Skin problems (e.g., rashes, hives, red skin,	58	63	121
tingling skin, dermatitis)	19.00%	7.60%	10.60%
Cognitive problems (e.g., difficulties thinking,	43	23	66
concentrating, or remembering)	14.10%	2.80%	5.80%
Mucosal symptoms (e.g., watery or red eyes, nasal	83	101	184
congestion, sneezing)	27.20%	12.10%	16.20%
Immune system problems (e.g., swollen lymph	34	11	45
glands, fever, fatigue)	11.10%	1.30%	4.00%
Gastrointestinal problems (e.g., nausea, bloating,	37	26	63
cramping, diarrhea)	12.10%	3.10%	5.50%
Cardiovascular problems (e.g., fast or irregular	30	20	50
heartbeat, jitteriness, chest discomfort)	9.80%	2.40%	4.40%
Musculoskeletal problems (e.g., muscle or joint	29	14	43
pain, cramps, weakness)	9.50%	1.70%	3.80%
Other	4	15	19
	1.30%	1.80%	1.70%

Table KK

Demographics.

People who answer “Yes” To One Or More Of These Questions: Q2/Q3/Q4/Q5/Q6 (health problems from fragranced products)

Percentages relative to column totals

	Asthmatic	Non-asthmatic	General Population
Total	196	198	394
	100.00%	100.00%	100.00%
**Male/Female**			
Male	94	79	173
	48.00%	39.90%	43.90%
Female	102	119	221
	52.00%	60.10%	56.10%
**Age**			
18-24	20	14	34
	10.20%	7.10%	8.60%
25-34	53	44	97
	27.00%	22.20%	24.60%
35-44	59	57	116
	30.10%	28.80%	29.40%
45-54	44	41	85
	22.40%	20.70%	21.60%
55-65	20	42	62
	10.20%	21.20%	15.70%
65	-	-	-
	-	-	-
**USA Region**			
Northeast	36	27	63
	18.40%	13.60%	16.00%
Midwest	44	42	86
	22.40%	21.20%	21.80%
South	59	76	135
	30.10%	38.40%	34.30%
West	57	53	110
	29.10%	26.80%	27.90%

Table LL

Demographics.

People who answer “Yes” To One Or More Of These Questions: Q2/Q3/Q4/Q5/Q6 (health problems from fragranced products)

Percentages relative to rows for asthmatics, non-asthmatics, and general population (Table OO)

	Asthmatic	Non-asthmatic	General Population
Total	196	198	394
	64.26%	23.80%	34.65%
**Male/Female**			
Male	94	79	173
	69.12%	20.31%	32.95%
Female	102	119	221
	60.36%	60.10%	36.11%
**Age**			
18-24	20	14	34
	47.62%	16.87%	27.20%
25-34	53	44	97
	69.74%	23.28%	36.60%
35-44	59	57	116
	69.41%	27.67%	39.86%
45-54	44	41	85
	61.97%	22.65%	33.73%
55-65	20	42	62
	64.52%	24.28%	30.39%
65			
**USA Region**			
Northeast	36	27	63
	62.07%	18.12%	30.43%
Midwest	44	42	86
	70.97%	22.83%	34.96%
South	59	76	135
	56.19%	23.97%	31.99%
West	57	53	110
	71.25%	29.12%	41.98%

Table MM

Demographics.

People who answer “Yes” To One Or More Of These Questions: Q2/Q3/Q4/Q5/Q6 (health problems from fragranced products)

Percentages relative to column totals

	Asthmatic	Non-asthmatic	General Population
Total	196	198	394
	100.00%	100.00%	100.00%
**Male/Female**			
All Males	94	79	173
	48.00%	39.90%	43.90%
All Females	102	119	221
	52.00%	60.10%	56.10%
**Gender vs Age**			
Male 18-24	8	6	14
	4.10%	3.00%	3.60%
Male 25-34	30	17	47
	15.30%	8.60%	11.90%
Male 35-44	31	24	55
	15.80%	12.10%	14.00%
Male 45-54	17	15	32
	8.70%	7.60%	8.10%
Male 55-65	8	17	25
	4.10%	8.60%	6.30%
Female 18-24	12	8	20
	6.10%	4.00%	5.10%
Female 25-34	23	27	50
	11.70%	13.60%	12.70%
Female 35-44	28	33	61
	14.30%	16.70%	15.50%
Female 45-54	27	26	53
	13.80%	13.10%	13.50%
Female 55-65	12	25	37
	6.10%	12.60%	9.40%

Table NN

Demographics.

People who answer “Yes” To One Or More Of These Questions: Q2/Q3/Q4/Q5/Q6 (health problems from fragranced products)

Percentages relative to rows for asthmatics, non-asthmatics, and general population (Table OO)

	Asthmatic	Non-asthmatic	General Population
Total	196	198	394
	64.30%	23.80%	34.70%
**Male/Female**			
All Males	94	79	173
	69.10%	20.30%	33.00%
All Females	102	119	221
	60.40%	26.90%	36.10%
**Gender vs Age**			
Male 18-24	8	6	14
	50.00%	19.40%	29.80%
Male 25-34	30	17	47
	83.30%	18.10%	36.20%
Male 35-44	31	24	55
	73.80%	25.50%	40.40%
Male 45-54	17	15	32
	56.70%	19.20%	29.60%
Male 55-65	8	17	25
	66.70%	18.50%	24.00%
Female 18-24	12	8	20
	46.20%	15.40%	25.60%
Female 25-34	23	27	50
	57.50%	28.40%	37.00%
Female 35-44	28	33	61
	65.10%	29.50%	39.40%
Female 45-54	27	26	53
	65.90%	25.20%	36.80%
Female 55-65	12	25	37
	63.20%	30.90%	37.00%

Table OO

Demographics by gender versus age

	Asthmatic	Non-asthmatic	General Population
Total	305	832	1137
	100.00%	100.00%	100.00%
**Male/Female**			
All Males	136	389	525
	44.60%	46.80%	46.20%
All Females	169	443	612
	55.40%	53.20%	53.80%
**Gender vs Age**			
Male 18-24	16	31	47
	5.20%	3.70%	4.10%
Male 25-34	36	94	130
	11.80%	11.30%	11.40%
Male 35-44	42	94	136
	13.80%	11.30%	12.00%
Male 45-54	30	78	108
	9.80%	9.40%	9.50%
Male 55-65	12	92	104
	3.90%	11.10%	9.10%
Female 18-24	26	52	78
	8.50%	6.30%	6.90%
Female 25-34	40	95	135
	13.10%	11.40%	11.90%
Female 35-44	43	112	155
	14.10%	13.50%	13.60%
Female 45-54	41	103	144
	13.40%	12.40%	12.70%
Female 55-65	19	81	100
	6.20%	9.70%	8.80%
